# Infiltrated pre-adipocytes increase prostate cancer metastasis *via* modulation of the miR-301a/androgen receptor (AR)/TGF-β1/Smad/MMP9 signals

**DOI:** 10.18632/oncotarget.3619

**Published:** 2015-04-08

**Authors:** Hongjun Xie, Lei Li, Guodong Zhu, Qiang Dang, Zhenkun Ma, Dalin He, Luke Chang, Wenbing Song, Hong-Chiang Chang, John J. Krolewski, Kent L. Nastiuk, Shuyuan Yeh, Chawnshang Chang

**Affiliations:** ^1^ Sex Hormone Research Center, Department of Urology, The First Affiliated Hospital, Xi'an Jiaotong University, Xi'an, China; ^2^ George Whipple Lab for Cancer Research, Departments of Pathology and Urology, and The Wilmot Cancer Center, University of Rochester Medical Center, Rochester, New York, USA; ^3^ Department of Pathology and Laboratory Medicine, University of Rochester Medical Center, Rochester, New York, USA; ^4^ Sex Hormone Research Center, China Medical University/Hospital, Taichung, Taiwan

**Keywords:** prostate cancer, pre-adipocyte, mirna-301a, androgen receptor

## Abstract

High fat dietary intake may increase the risk of prostate cancer (PCa). Pre-adipocytes, one of the basic components in the tumor microenvironment (TME), are capable of differentiating into adipose tissues and play key roles to affect PCa progression. Here we found the pre-adipocytes could be recruited more easily to PCa than its surrounding normal prostate tissue. *In vitro* co-culture system also confirmed PCa has a better capacity than normal prostate to recruit pre-adipocytes. The consequences of recruiting more pre-adipocytes may then increase PCa cell invasion. Mechanism dissection revealed infiltrating pre-adipocytes might function through down-regulation of the androgen receptor (AR) *via* modulation of miR-301a, and then increase PCa cell invasion *via* induction of TGF-β1/Smad/MMP9 signals. The mouse model with orthotopically xenografted PCa CWR22Rv1 cells with pre-adipocytes also confirmed that infiltrating pre-adipocytes could increase PCa cell invasion *via* suppressing AR signaling. Together, our results reveal a new mechanism showing pre-adipocytes in the prostate TME can be recruited to PCa to increase PCa metastasis *via* modulation of the miR-301a/AR/TGF-β1/Smad/MMP9 signals. Targeting this newly identified signaling may help us to better inhibit PCa metastasis.

## INTRODUCTION

Prostate cancer (PCa) is the second most commonly diagnosed cancer in males worldwide [1]. Androgen receptor (AR) signaling plays critical roles for PCa initiation and progression [2, 3], and previous studies showed that down-regulation of AR with si-AR or IL-6 could enhance PCa cell invasion [4, 5]. Early studies suggested that individual cells within the prostate tumor microenvironment (TME), including macrophages [5–7], endothelial cells [4] and bone marrow mesenchymal stem cells (BM-MSCs) [8] might influence the PCa progression, however, very few studies show pre-adipocyte cells are involved in PCa initiation and progression.

Obesity has been suggested to be a risk factor for PCa [9, 10], and pre-adipocytes are capable of proliferating and differentiating into an adipose deposit to play a key role in the progression of obesity [11], and influence various tumor progressions [12–15]. However, results from those studies indicated that pre-adipocytes had controversial effects on tumor progression including simulating breast cancer and uterine leiomyoma growth [12, 14], yet inhibiting breast cancer proliferation and metastasis [13, 15]. The potential impact of pre-adipocytes on PCa progression, however, remains unclear.

Micro-RNAs (miRNAs), a new class of small RNAs, were found to be able to influence the tumor progression *via* modulating both mRNA stability and the mRNA translation ability into protein [16, 17]. The newly identified microRNA, miR-301a, is associated with breast cancer and gastric cancer progression [18, 19].

Our studies found recruited pre-adipocytes could enhance PCa cell invasion *via* modulation of miR-301a-AR signaling.

## RESULTS

### Prostate cancer recruits more pre-adipocytes than normal prostate tissues

**Figure 1:**
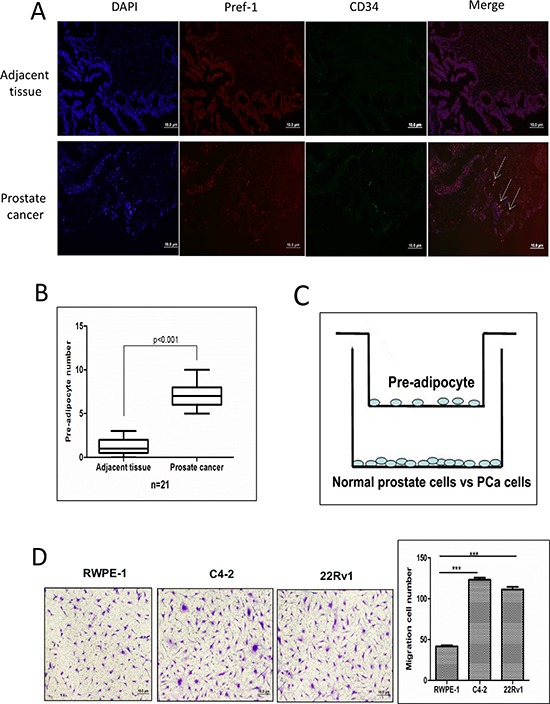
Prostate cancer recruits more pre-adipocytes than normal prostate **A.** Pref-1 and CD34 staining to show pre-adipocyte distribution in human PCa and adjacent normal tissues by immunofluorescence co-staining. **B.** Quantification of pre-adipocyte numbers in PCa and normal prostate tissues. **C.** Cartoon illustration of the pre-adipocyte recruitment to PCa and normal prostate epithelial cells experimental procedure. Pre-adipocyte cells (5 × 10^4^) were placed in the upper chambers and the PCa cells were cultured in the bottom chambers to assay the migration of pre-adipocyte cells. After 24 hours, the membranes were fixed and stained to visualize the migrated pre-adipocyte cells on the bottom of the membrane. **D.** The representative figures and quantitative data of pre-adipocyte recruitment migration by normal prostate cell, RWPE-1, and PCa C4-2 and CWR22Rv1 (22Rv1) cells. ***p < 0.005.

We first applied immunofluorescence co-staining using pre-adipocyte markers Pre-adipocyte factor-1 (Pref-1) and CD34 to examine the distribution of pre-adipocytes (double positive) in human PCa samples [20, 21], and results revealed that there were more pre-adipocytes in the PCa area compared to the adjacent normal prostate tissues (Fig. 1A-B). We also co-stained these two pre-adipocytes markers in human primary pre-adipocytes from ATCC to confirm the presence of these two markers, and we found they are both 100% positive in human primary pre-adipocytes (Supplementary Fig. S1).

To confirm these *in vivo* clinical staining data, we then applied the Boyden chamber migration system to assay the pre-adipocyte migration ability to PCa cells vs normal prostate epithelial cells (see cartoon in Fig. 1C). As shown in Fig. 1D, PCa C4-2 cells have better capacity to recruit more human primary pre-adipocytes than human normal prostate RWPE-1 cells. Similar results were also obtained when we replaced C4-2 PCa cells with CWR22Rv1 cells (Fig. 1D) or with the mouse 3T3-L1 pre-adipocyte cell line (Supplementary Fig. S2).

Together, results from both human clinical data (Fig. 1A-B) and *in vitro* cell co-culture data (Fig. 1C-D) demonstrated that PCa cells could recruit more pre-adipocytes than normal prostate cells.

### Enhanced pre-adipocyte recruitment increased PCa cell invasion

**Figure 2:**
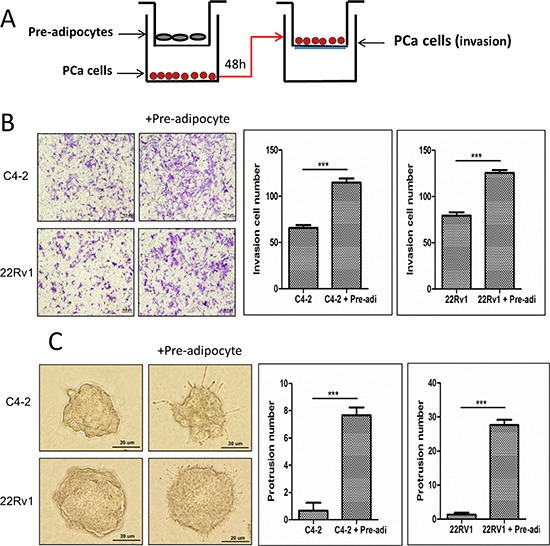
Increased pre-adipocyte recruitment could promote PCa cell invasion **A.** The cartoon illustrates the co-culture system. We co-cultured PCa cells with pre-adipocytes for 2 days, then the trypsinized PCa cells were seeded in Boyden chamber invasion system upper chambers pre-coated with Matrigel to perform invasion assay. **B.** Image shows PCa cells co-cultured with pre-adipocytes (pre-adi) have a higher invasiveness. The right panels are the quantification data of changed PCa invasion abilities. **C.** 3D invasion assay results showed PCa cells have more protrusions after co-culture with pre-adipocytes (pre-adi). ***p < 0.005.

We then applied the Boyden Chamber invasion assays in the co-culture system (Fig. 2A) to examine the consequences of increased infiltrating pre-adipocytes to PCa. The results revealed that PCa (C4-2 and CWR22Rv1) cells, after co-culture with pre-adipocytes, become more invasive in the Boyden chamber invasion system (Fig. 2B). Similar results were also obtained when we replaced human primary pre-adipocytes with the mouse 3T3-L1 pre-adipocyte cell line (Supplementary Fig. S3A).

Importantly, using another 3D culture invasion assay, we also obtained similar results showing the PCa C4-2 and CWR22Rv1 cells had better invasive ability after co-culture with pre-adipocytes (Fig. 2C). Similar results were also obtained when we replaced human primary pre-adipocytes with mouse 3T3-L1 pre-adipocyte cell line (Supplementary Figure S3B).

### Recruited pre-adipocyte enhanced PCa cell invasion *via* alteration of AR/TGF-β1/Smad/MMP9 signals

**Figure 3:**
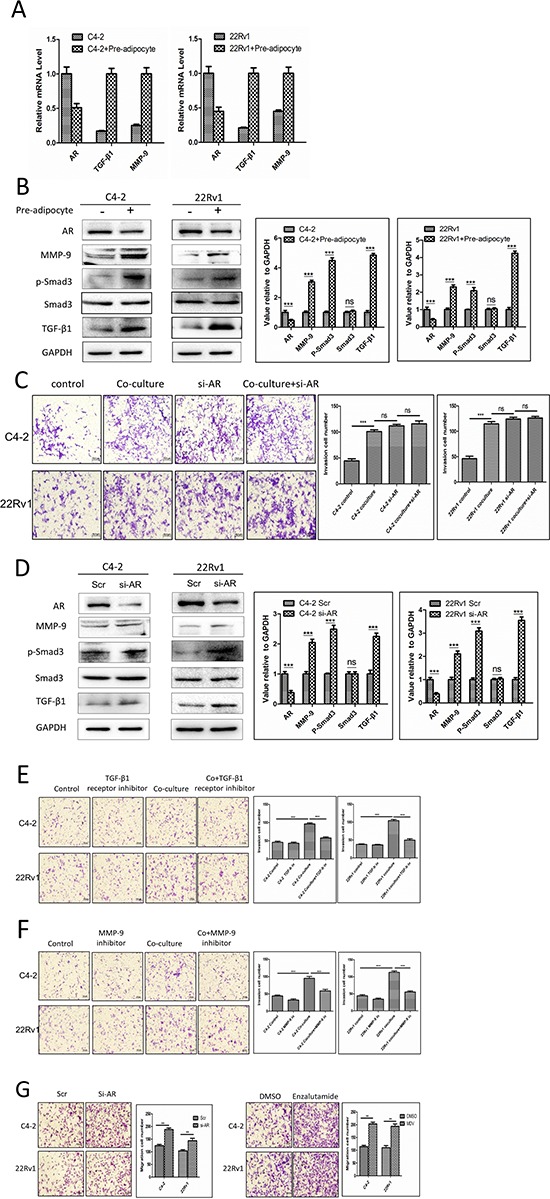
Recruited pre-adipocytes enhanced PCa cell invasion *via* alteration of AR/TGF-β1/Smad/MMP9 signaling **A.** mRNA level shows AR is down-regulated, TGF-β1 and MMP-9 are up-regulated after co-culture with pre-adipocytes (pre-adi). **B.** AR protein level is down-regulated, TGF-β1, p-Smad3 and MMP-9 protein levels are up-regulated in PCa cells after co-culture with pre-adipocytes (pre-adi). The right panels are the quantitative data for western-blot. ***p < 0.005, ns, no statistical differences. **C.** The representative and quantitative invasion abilites of PCa cells after co-culture with pre-adipocytes following knocking-down PCa cells AR. ***p < 0.005, ns, no statistical differences. **D.** Knocking down AR in PCa cells can decrease AR protein levels, and increase TGF-β1, p-Smad3 and MMP-9 protein levels. The right panels are the quantitative data for western-blot. ***p < 0.005, ns, no statistical differences. **E.** TGF-β1 type I receptor inhibitor, SB-431542 interrupts pre-adipocyte mediated PCa invasion. **F.** Targeting MMP9 by specific inhibitor interrupts pre-adipocyte mediated PCa invasion. **G.** The recruitment effect for pre-adipocytes by knocking down AR or adding anti-androgen enzalutamide in C4-2 and CWR22Rv1 (22Rv1) cells.

To dissect the molecular mechanisms why increased infiltrating pre-adipocytes could enhance PCa cells invasion, we focused on the influence of the AR, the key player controlling PCa cells invasion [2, 3, 5, 7]. As shown in Fig. 3A-B, the recruitment of pre-adipocytes to PCa cells decreased AR expression at both mRNA (Fig. 3A) and protein (Fig. 3B) levels.

We also assayed the recruited pre-adipocytes impacts on AR downstream metastasis-related target genes including the TGF-β1 and MMP-9, which are negatively regulated by AR [4]. As shown in Fig. 3A-B, the recruitment of pre-adipocytes to PCa cells also increased TGF-β1, p-Smad3 and MMP-9 expression at both mRNA and protein levels in C4-2 and CWR22Rv1 cells. Similar results were also obtained when we replaced human primary pre-adipocytes with mouse 3T3-L1 pre-adipocyte cell line at the mRNA level (Supplementary Fig. S4).

To further confirm these conclusions, we applied the interruption approaches knocking-down AR in C4-2 and CWR22Rv1 cells, and found knocking-down AR could enhance the PCa cell invasion, importantly, knocking-down AR in C4-2 and CWR22Rv1 interrupted the infiltrated pre-adipocytes activity to further enhance PCa invasion (Fig. 3C), and importantly, knocked-down AR could then increase the expression of TGF-β1, p-Smad3 and MMP-9 expression (Fig. 3D), suggesting down-regulation of AR may play essential roles for the infiltrated pre-adipocytes to increase the expression of TGF-β1, p-Smad3 and MMP-9 expression to impact the PCa progression.

We then applied similar interruption approaches with the inhibitor of TGF-β type I receptor (SB-431542) and MMP-9 specific inhibitor to suppress the AR/TGF-β1/Smad/MMP9 signals, and results revealed that both of the inhibitors could partially reverse the pre-adipocyte-enhanced cell invasion in both C4-2 and CWR22Rv1 cells (Fig. 3E-F).

Together, results from Fig. 3A-F suggest that the recruitment of pre-adipocytes to PCa could enhance the PCa cell invasion *via* modulation of the AR/TGF-β1/Smad/MMP9 signals in PCa cells.

### Feed-back mechanism for AR to influence the recruitment of pre-adipocytes to PCa

Interestingly, we found suppressed AR signals promoted the PCa cell invasion, but could also enhance the PCa cells ability to recruit more pre-adipocytes. As shown in Figure 3G, targeting AR with either AR-siRNA or the anti-androgen enzalutamide led to recruiting more pre-adipocytes in the co-culture system. These results suggested the existence of a positive feed-back mechanism, the suppression of PCa AR after recruiting pre-adipocytes could then recruit more pre-adipocytes, which might then further suppress AR signals. The consequence of this feed-back mechanism might then significantly enhance the PCa cell invasion.

### Mechanism dissection how infiltrating pre-adipocytes could suppress PCa AR expression

**Figure 4:**
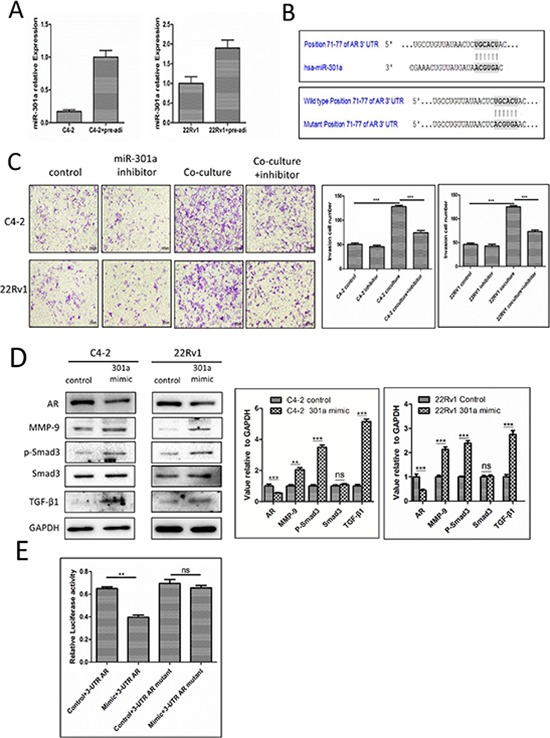
Mechanisms why infiltrating pre-adipocytes could suppress PCa AR expression to promote PCa cell invasion **A.** miR-301a increased in PCa cells after co-culture with pre-adipocytes (pre-adi). **B.** Predicted duplex formation between human miR-301a and human AR 3′UTR (top panel). Mutant site of 3′UTR of AR (bottom panel). **C.** PCa cells were transfected with miRNA-301a inhibitor co-cultured with pre-adipocytes, then the invasion assay was performed. **D.** The AR and its downstream changes in PCa cells transfected with miRNA-301a. The right panels are the quantitative data for western-blot. **p < 0.01, ***p < 0.005, ns, no statistical differences. **E.** C4-2 cells transfected with miRNA-301a, wild type 3′UTR of AR reporter PGL-3 plasmid or mutant 3′UTR of AR reported PGL-3 plasmid to perform the luciferase assay. **p < 0.05, ns, no statistical differences.

To dissect the mechanism how recruited pre-adipocytes could suppress the PCa AR expression, we focused on those reported miRNAs that were able to modulate the AR expression. We screened some of these miRNAs with the TargetScan programs to search for miRNAs that target evolutionary conserved sequences in the 3′UTR of the AR, and we found the expression of miRNA-301a was increased in both PCa cell lines C4-2 and CWR22Rv1 after co-culture with pre-adipocytes (Fig. 4A-B, Supplementary Fig. S5).

We then applied the interruption approach using miRNA-301a inhibitor treated PCa cells and results revealed that it could partially reverse pre-adipocytes capacity to promote PCa invasion (Fig. 4C), and importantly, addition of miRNA-301a to the PCa cells also show the similar modulation of the AR/TGF-β1/Smad/MMP-9 signals as pre-adipocytes co-cultured with PCa cells (Fig. 4D).

We then examined how miRNA-301a targets the AR with construction of the wild-type 3′UTR of AR reporter plasmid and mutant 3′UTR of AR reporter plasmid (See Fig. 4B) for the luciferase assay, and results revealed that miRNA-301a could significantly suppress the 3′UTR of AR luciferase activity (Fig. 4E).

Together, results from Figure 4A-E suggest that recruited pre-adipocytes may function through modulation of miRNA-301a to suppress AR to promote the PCa cell invasion in C4-2 and CWR22Rv1 cells.

### Pre-adipocytes promote PCa invasion using mouse PCa model

**Figure 5:**
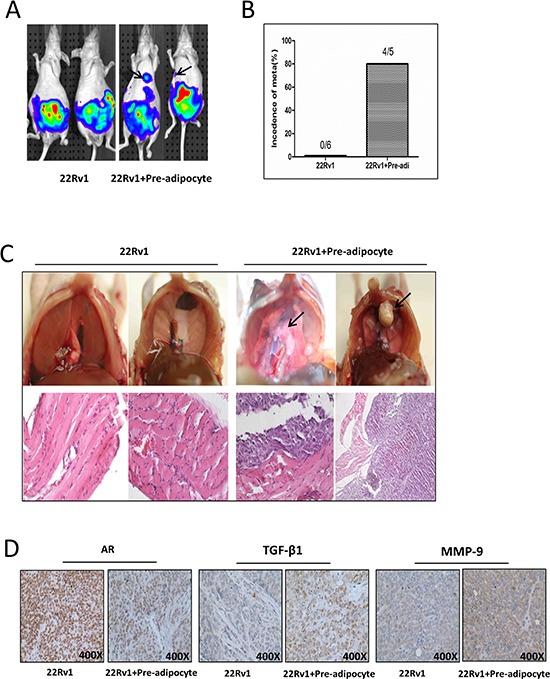
Pre-adipocytes promote PCa invasion using *in vivo* orthotopic PCa model **A.** Orthotopic-implantation of human immortalized pre-adipocytes together with PCa promotes PCa invasion cells. The CWR22Rv1 (22Rv1) cells were transfected with pCDNA-luciferase. 22Rv1-luc cells (1 × 10^6^) were mixed with or without pre-adipocytes (1 × 10^5^) and orthotopically implanted into the AP of nude mice. After 6 weeks implantation, the PCa growth was monitored by IVIS images. Arrows show the metastatic foci. **B.** Quantification data for tumor metastases in mice. **C.** Image illustrates metastasized tumors in the diaphragm. The top panels show the tumor mass on diaphragm, and the bottom panels are the H&E staining to confirm the tumor mass is cancer. Arrows show the metastatic foci. **D.** IHC staining for AR, TGF-β1 and MMP-9 in orthotopic mice tumor tissues.

To demonstrate all above *in vitro* cell lines results in the *in vivo* animal models, we then orthotopically xenografted PCa cells into the anterior prostates (AP) of mice using CWR22Rv1 cells stably transfected with pCDNA-luciferase to monitor the PCa progression with IVIS Imaging system [8]. After xenografting 1 × 10^6^ PCa CWR22Rv1-luc cells with or without mixed 1 × 10^5^ human immortalized pre-adipocytes for six weeks, we found (from IVIS image) metastatic foci, marked by arrows (Fig. 5A), in 4 out of 5 mice with co-implanted PCa and pre-adipocyte cells. In contrast, no metastatic foci were found in mice with PCa CWR22Rv1-luc cells (Fig. 5A-B). These results from IVIS image were further confirmed showing identified metastatic foci in the diaphragm and H&E staining after mice were sacrificed (Fig. 5C).

Importantly, results from IHC staining of related key factors including AR, TGF-β1 and MMP-9 also matched well with those data from *in vitro* co-culture studies (Fig. 5D).

## DISCUSSION

More than 30% of adults in the United States are obese [22]. Obesity has been linked to several types of cancer, including postmenopausal breast and colon cancers [23]. Several large studies also indicated that increased BMI was associated with increased risk of PCa [24, 25].

Pre-adipocytes are capable of proliferating and differentiating into an adipose deposit [11]. Stimulation of the proliferation of these cells may therefore result principally in an increase in adipocyte numbers leading to obesity. Interestingly, pre-adipocyte roles in tumor progression still remain controversial [13–15], and their roles in PCa progression also remain unclear. Here we found the recruitment of pre-adipocytes by PCa cells could further enhance PCa cell invasion, which might represent the first such finding.

Mechanism dissection found pre-adipocytes might function through modulation of AR signaling to enhance PCa cell invasion, which is in agreement with early studies showing targeting AR with AR-siRNA might lead to enhance PCa cell invasion [4]. Other studies also indicated that some miRNAs could target AR to influence PCa progression. For example, miR-124 could directly target AR and subsequently induce p53 expression to influence the PCa progression [26]. MiR-185 was also found to be down-regulated in clinical PCa samples and could reduce the AR expression to suppress LNCaP cell growth [27]. Infiltrating T cells to PCa could also suppress AR expression *via* miR-541 to influence PCa cell invasion [28].

Here we linked the miR-301a as an upstream molecule that modulates AR expression *via* targeting the 3′UTR of AR to inhibit its translation. Early studies indicated that miR-301a might function as a tumor promoter to alter the progression of many tumors, including breast cancer, pancreatic cancer, hepatocellular carcinoma, gastric cancer and colorectal cancer [18, 19, 29–32]. Mechanism dissection indicated that the miR301a promoted breast cancer proliferation and metastasis *via* targeting FOXF2, BBC3, PTEN, and COL2A1 [18], as well as down-regulated NF-kB-repressing factor and elevated NF-kB activation in human pancreatic adenocarcinoma [29]. Our finding that infiltrating pre-adipocytes could induce miR-301a to promote PCa metastasis *via* down-regulating AR, represents another new mechanism for miR-301a to alter the tumor progression.

Further mechanism dissection also found AR could alter the TGFβ-1 and its downstream genes, Smad3 and MMP-9 signals to influence the PCa cell invasion. Down-regulation of AR could increase TGF-β1 expression *via* transcriptional regulation, which has been reported previously [33]. Early studies indicated that TGF-β1 signaling is mediated through two types of transmembrane serine/threonine kinase receptors [34], and the activated TGF-β receptors might then interact with an adaptor protein SARA (Smad anchor for receptor activation) [35], which propagates signals to the intracellular signaling mediators Smad2 and Smad3 [36]. Following association with Smad4, the Smad complexes then translocate to the nuclei, where they activate specific target genes including MMP-9 through cooperative interactions with its DNA and other DNA-binding proteins to control tumor progression [4, 37].

**Figure 6:**
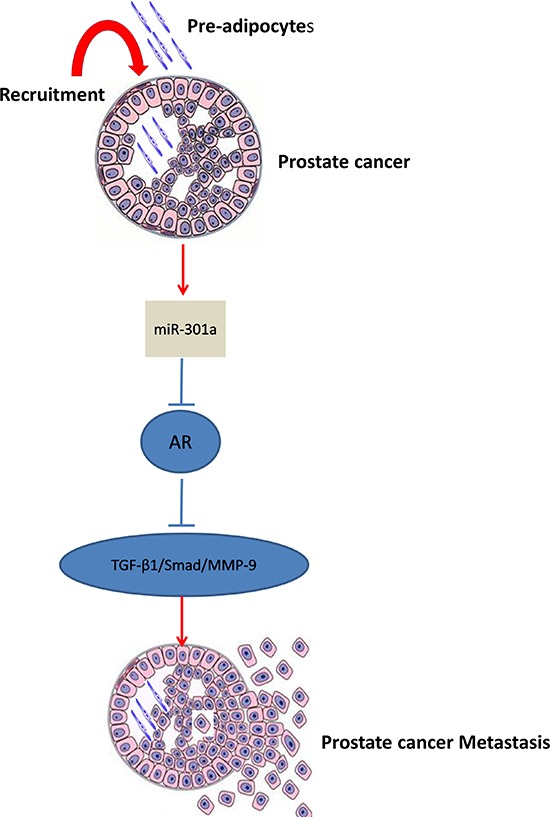
Mechanisms and regulatory pathways of Pre-adipocyte-promoted PCa invasion Pre-adipocytes can down-regulate androgen receptor (AR) in PCa cells *via* increase of miRNA-301a, then activate TGF-β1/Smad/MMP-9 pathway.

In summary, our results conclude that infiltrating pre-adipocytes could promote PCa metastasis *via* modulation of the miR-301a/AR/TGF-β1/Smad/MMP9 signaling (Fig. 6), which might explain the link of potential obesity influences on the PCa progression. Future studies using small molecules to target this newly identified signaling may provide us a new potential therapeutic approach to better battle PCa metastasis.

## MATERIALS AND METHODS

### Human specimens

Tumor specimens and adjacent normal prostate tissues were collected from a total of 21 patients that showed clinical evidence of PCa by biopsy or treated with radical prostatectomy at the first Affiliated Hospital, Xi'an Jiaotong University, China. All specimens were obtained on the basis of their availability for research purposes and under a protocol approved by the local Medical Ethics Committee of the First Affiliated Hospital, Xi'an Jiaotong University, China.

### Cell lines

The CWR22Rv1 cells, human primary pre-adipocyte cell line and mouse pre-adipocyte cell line 3T3-L1 were purchased from the American Type Culture Collection (Rockville, MD). CWR22Rv1 cells were cultured in RPMI 1640 with 10% FBS. Human primary pre-adipocyte cell line was maintained in Fibroblast Basal Medium Containing 1% penicillin and streptomycin, supplemented with Fibroblast Growth Kit-low serum components (ATCC^®^PSC-201-041). 3T3-L1 cells were cultured in DMEM supplemented with 10% FBS (v/v). The immortalized human primary pre-adipocyte cell line was infected with SV40 lentivirus and maintained in Fibroblast Basal Medium. The C4-2 cell line was a gift from Dr. Jer-Tsong Hsieh (Southwestern Medical Center) and grown in RPMI-1640 media containing 1% penicillin and streptomycin, supplemented with 10% FBS. The immortalized non-transformed RWPE-1 prostate epithelial cell line was purchased from American Type Culture Collection and grown in keratinocyte serum free medium (K-SFM) supplemented with bovine pituitary extract (BPE) and human recombinant epidermal growth factor (EGF).

### Reagents and materials

GAPDH (6c5), Pref-1, CD34, TGF-β1 and AR (N-20) antibodies were purchased from Santa Cruz Biotechnology (Paso Robles, CA). p-Smad3 and Smad3 antibodies were purchased from Cell Signaling Technology Company (Boston, MA), MMP-9 (ab38898) antibody was from Abcam Company (San Diego, CA). TGF-β type I receptor inhibitor (SB-431542) and MMP-9 specific inhibitor were from Cell Signaling Technology Company (Boston, MA). Crystal violet was from Fishers Scientific Company (Grand Island, NY). Anti mouse/rabbit second antibody for Western Blot and Lipofectamine 3000 transfection reagent were purchased from Life Technologies Company (Grand Island, NY). MiRNA-301a mimics and inhibitors were purchased from QIAGEN (Valencia, CA).

### Immunofluorescence microscopy

The tumor samples from prostate tumors in situ were fixed in 4% neutral buffered para-formaldehyde and embedded in paraffin. Prostate sections were deparaffinized in xylene solution and rehydrated using gradient ethanol concentrations. Sections were antigen retrieved and washed, blocked in PBS containing 5% BSA for 1 hr at room temperature in a humidified chamber. Sections were washed again and incubated with Pref-1 antibody (mouse) and CD34 (Rabbit) overnight at 4°C. Sections were washed with PBS and incubated with Invitrogen Alexa Fluor 488 goat anti-mouse IgG antibody (1:500) and Alexa Fluor 594 goat anti-rabbit IgG antibody (1:500) for 1 hr in the dark at room temperature. Slides were washed with PBS, stained with DAPI for 5 min, added with Thermal mountant permafluor and sealed with cover glasses. Sections were observed under fluorescence microscope and images were captured.

### Pre-adipocyte recruitment assay

Pre-adipocytes migration was detected using 24-well transwell assays. Briefly, PCa cells were placed in the lower chambers of 24-well transwells. Pre-adipocytes (5 × 10^4^ cells/well) were then seeded in the upper chambers. The upper and lower chambers were separated by an 8 μm polycarbonate membrane. The chambers were incubated for 24 hours at 37°C, then washed, fixed with cold methanol, and stained with 0.1% crystal violet. Cell migration was measured by counting the number of cells attached to the lower surface of the membrane. Each PCa cell line was tested in triplicate. The results were expressed as the mean of the number of migrating cells.

### Cell invasion assay

24-well (8 μm pores) transwell plates (Corning, Lowell, MA) were used for invasion assay. For *in vitro* invasion assays, the upper chambers of the transwells (8 μm) were precoated with diluted matrigel (BD Biosciences, Sparks, MD). Before performing invasion assays, PCa cells were cocultured with pre-adipocytes for 48 hrs and then trypsinized. 10^5^ PCa cells (in serum free media) and media containing 10% serum were plated in the upper and lower chambers, respectively. After 48 hrs incubation, invaded cells were stained with 0.1% crystal violet, and positively stained cells were counted. The cell numbers were calculated from counting six random fields. Quantitation indicates mean ± SEM of triplicate repeats.

### 3D invasion assay

Thaw Matrigel on ice and add 40 μl to each well of 8-well glass chamber slides (at 50 μl/cm^2^) and spread the Matrigel evenly, place the slides in the cell culture incubator and allow the Matrigel to solidify (takes 15–20 min). Plate 1 × 10^4^ C4-2 and CWR22Rv1 cells after co-culture with pre-adipocytes into each well with media containing 5% Matrigel and 10 ng/ml EGF. Change media once every 3 days with assay media containing 2.5% Matrigel and 5.0 ng/ml EGF. PCa cells take about 7 days to form protrusion structures. 10 different fields under 200x microscope were chosen randomly to count the number of protrusions on cells in each field.

### Luciferase constructs

To construct luciferase reporter plasmids, we inserted various target fragments into multiple cloning sites (MCS; SacI and HindIII) downstream of the Renilla luciferase reporter gene in the PGL-3 promoter vector (Promega, Madison, WI). 5′ Modified primers carrying restriction sites for either SacI or HindIII were used. The 3000-nt fragment of the AR 3′UTR was generated using the following primer sets: forward, 5′-CTA GTC AGA TGT CTT CTG CCT GTT ATA ACT CAG CAC AAC TCC TCT GCA GTG CCT TGC CGG-3′ and reverse, 5′-CAA GGC ACT GCA GAG GAG TTG TGC TGA GTT ATA ACA GGC AGA AGA CAT CTG A-3′. Site-directed mutagenesis of the miR-301a binding site in the AR 3′UTR was carried out with the QuickChange Site-Directed Mutagenesis Kit (Stratagene, La Jolla, CA). A fragment containing the perfect matching sequence with the mature miR-301a was cloned. All constructs were verified *via* sequencing. We also constructed the miR-301a binding site mutant 3′UTR of AR reporter PGL-3 plasmid.

### MiRNA transfection

MiRNA mimic transfection was performed as previously reported. Briefly, 33 nM miRNA and 3 ul P3000 in 125 μl Opti-MEM^®^ I reduced serum media were transfected by Lipofectamine^®^ 3000 system (invitrogen), and then dilute 4 μl Lipofectamine^®^ 3000 in 125 μl Opti-MEM® I reduced serum media. Mix gently and incubate for 5 minutes at room temperature. After the 5 min incubation, combine the diluted miRNA with the diluted Lipofectamine^®^ 3000. Mix gently and incubate for 5 min at room temperature, and then add the mixture to the cells.

### Luciferase reporter assays

Cells were plated in 24-well plates and transfected with the 3′UTR of AR-luciferase pGL3, miRNA-301a mimics and pRL-TK-luciferase plasmid using Lipofectamine3000 (Invitrogen) according to the manufacturer's instructions. Cells were lysed and the luciferase activity was detected by the dual luciferase assay using pRL-TK-luciferase as the internal control. Each sample was normalized by pRL-TK-luciferase activity, and all data were presented as mean ± SEM from at least three independent experiments.

### RNA extraction and quantitative real-time PCR analysis

Total RNAs were isolated using Trizol reagent (Invitrogen). One μg of total RNA was subjected to reverse transcription using Superscript III transcriptase (Invitrogen). Quantitative real-time PCR (Q-RT-PCR) was conducted using a Bio-Rad CFX96 system with SYBR green to determine the mRNA expression level of a gene of interest. Expression levels were normalized to the expression of GAPDH RNA.

### Western blot analysis

Cells were lysed in RIPA buffer and proteins (20 μg) were separated on 8–10% SDS/PAGE gel and then transferred onto PVDF membranes (Millipore, Billerica, MA). After blocking membranes, they were incubated with appropriate dilutions (1:1000) of specific primary antibodies, the blots were incubated with HRP-conjugated secondary antibodies and visualized using ECL system (Thermo Fisher Scientific, Rochester, NY).

### *In vivo* metastasis studies

Male 6- to 8-week old nude mice were used. CWR22Rv1 cells were engineered to express luciferase reporter gene (PCDNA3.0-luciferase) by stable transfection and the positive stable clones were selected and expanded in culture. 6 mice were injected with 1 × 10^6^ PCa luciferase expressing cells, as a mixture with Matrigel, 1:1 and another 5 mice were co-injected with 1 × 10^6^ PCa cells and 1 × 10^5^ immortalized human pre-adipocytes to anterior prostate (AP). Metastasis in live mice was monitored using a Fluorescent Imager (IVIS Spectrum, Caliper Life Sciences, Hopkinton, MA) at different time points (3, 4, 5, and 6 wks after injection) following intraperitoneal injection of Luciferin. After a final monitoring with the Imager, mice were sacrificed and the metastases were further examined by H&E. All animal studies were performed under the supervision and guidelines of the University of Rochester Medical Center Animal Care and Use Committee.

### H&E staining

The tissue sections were de-waxed and rehydrated routinely. The sections were stained in hematoxylin for 5 min and washed in running tap water for 5 min. Then the sections were stained in eosin for 30 sec, dehydrated, and mounted by routine methods. The representative fields were chosen to present in the figures.

### Histology and IHC staining

Mouse prostate tissues were fixed in 10% (v/v) formaldehyde in PBS, embedded in paraffin, and cut into 5 μm sections. Prostate sections were deparaffinized in xylene solution and rehydrated using gradient ethanol concentrations, and immunostaining was performed.

### Statistics

All statistical analyses were carried out with SPSS 19.0 (SPSS Inc, Chicago, IL). The data values were presented as the mean ± SEM. Differences in mean values between two groups were analyzed by two-tailed Student's *t* test, and the means of more than two groups were compared with one way ANOVA. *p* ≤ 0.05 was considered statistically significant

## SUPPLEMENTARY FIGURES


