# Correction: Infiltrated pre-adipocytes increase prostate cancer metastasis *via* modulation of the miR-301a/androgen receptor (AR)/TGF-β1/Smad/MMP9 signals

**DOI:** 10.18632/oncotarget.13913

**Published:** 2016-12-12

**Authors:** Hongjun Xie, Lei Li, Guodong Zhu, Qiang Dang, Zhenkun Ma, Dalin He, Luke Chang, Wenbing Song, Hong-Chiang Chang, John J. Krolewski, Kent L. Nastiuk, Shuyuan Yeh, Chawnshang Chang

**Present**: Due to an error made during the assembly of Figure 3B, 3G and 4C. It has come to the authors’ attention that wrong figure panels in Figure 3B, 3G and 4C were provided. After checking the question figure, we found that, by mistake, during preparation of these multiple set of figure panels, some of the images that had been included were incorrect.

**Correct**: Correct Figure 3B, 3G and 4C is provided below. The authors sincerely apologize for this error.

Original article: Oncotarget. 2015; 6(14):12326-39. doi: 10.18632/oncotarget.3619.

**Figure 3:**
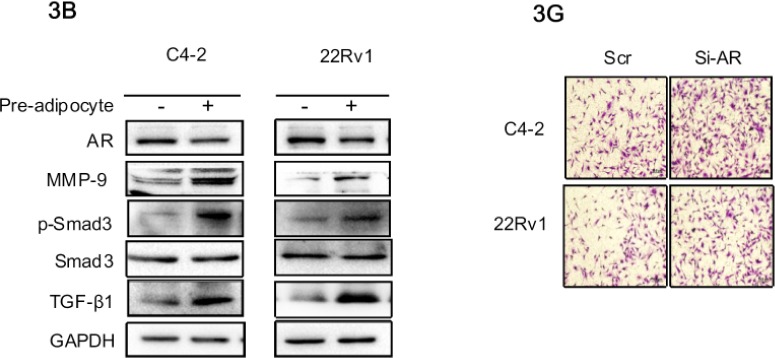
Recruited pre-adipocytes enhanced PCa cell invasion via alteration of AR/TGF-β1/Smad/MMP9 signaling B. AR protein level is down-regulated, TGF-β1, p-Smad3 and MMP-9 protein levels are up-regulated in PCa cells after co-culture with pre-adipocytes (pre-adi). The right panels are the quantitative data for western-blot. ***p < 0.005, ns, no statistical differences. G. The recruitment effect for pre-adipocytes by knocking down AR or adding anti-androgen enzalutamide in C4-2 and CWR22Rv1 (22Rv1) cells.

**Figure 4:**
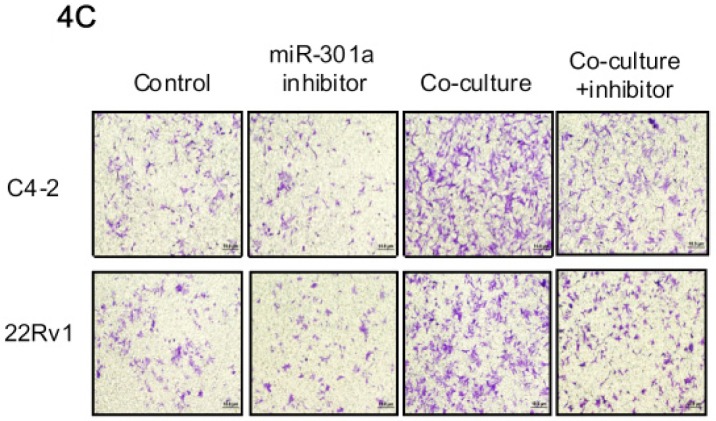
Mechanisms why infiltrating pre-adipocytes could suppress PCa AR expression to promote PCa cell invasion C. PCa cells were transfected with miRNA-301a inhibitor co-cultured with pre-adipocytes, then the invasion assay was performed.

