# Supplementary material for: Infiltrated pre-adipocytes increase prostate cancer metastasis via modulation of the miR-301a/androgen receptor (AR)/TGF-β1/Smad/MMP9 signals
Source: Oncotarget. 2015 Apr 8;6(14):12326–39. doi: 10.18632/oncotarget.3619 (PMC4494941; doi:10.18632/oncotarget.3619)
Supplement: Supplementary file 1 [file oncotarget-06-12326-s001.pdf]

SUPPLEMENTARY FIGURES

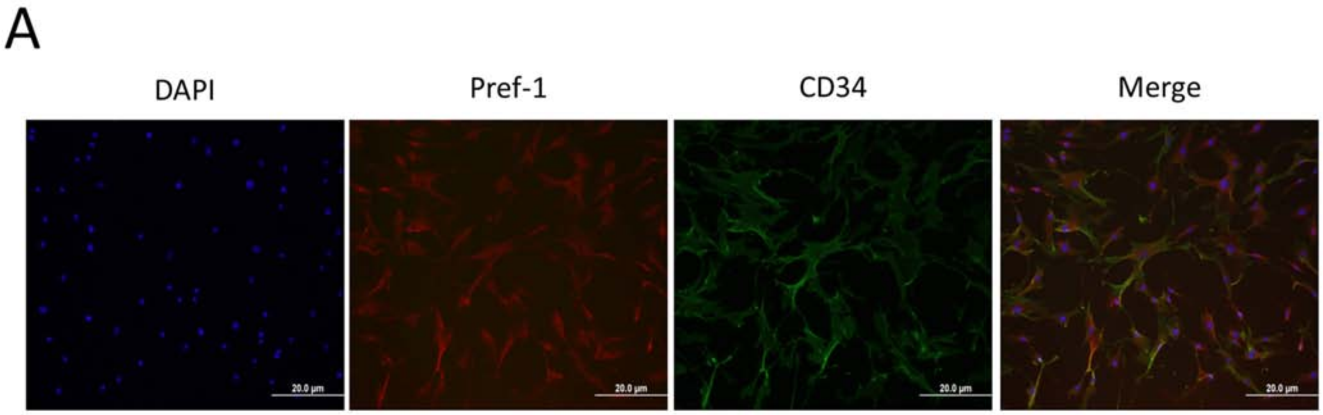

Supplementary Figure S1: Co-staining of CD34 and Pref-1 in human primary pre-adipocytes. The expression of CD34 and Pref-1 in human primary pre-adipocytes.

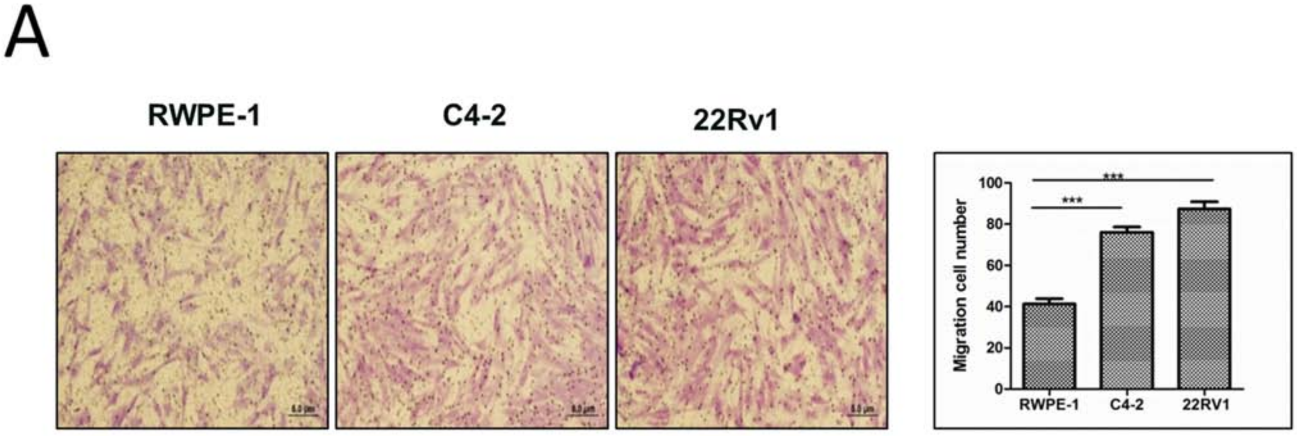

Supplementary Figure S2: Human PCa cells promote mouse pre-adipocytes 3T3-L1 cells recruitment migration. Mouse pre-adipocytes (5 × 104) were added in the upper well, and the non-malignant prostate RWPE-1 cells and PCa cell lines, C4-2 and CWR22Rv1 (22Rv1) (1 × 105) were seeded in lower wells to do recruitment migration assay for 24 hr. ***p < 0.005.

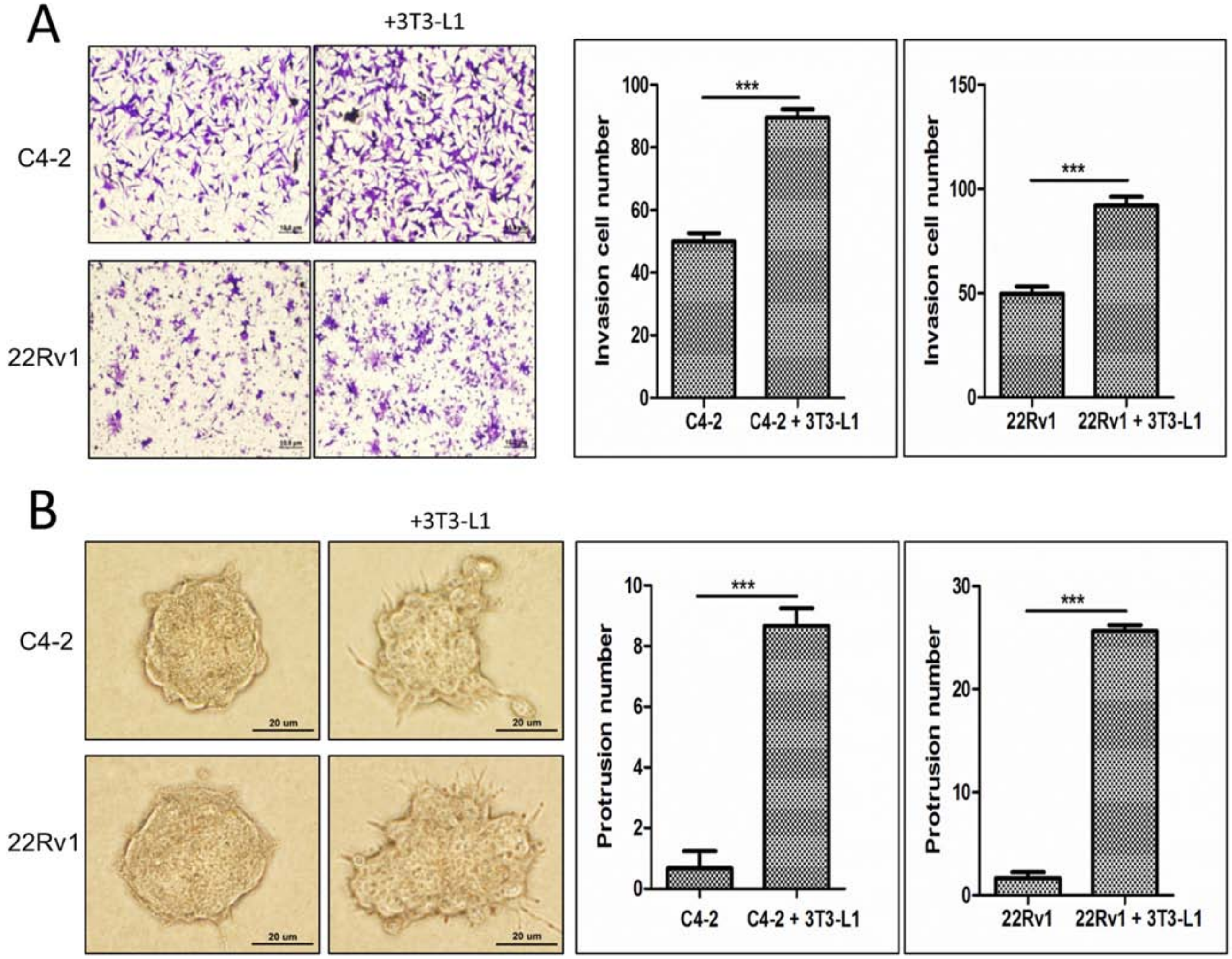

Supplementary Figure S3: Invasion assay of PCa cells after co-culture with mouse pre-adipocytes. A. Image shows PCa cells co-cultured with mouse pre-adipocytes 3T3-L1 cells have a higher invasiveness. The right panel is the quantification data of changed PCa invasion abilites. ***$p < 0.005$. **B.** 3D invasion assay results showed PCa cells have more protrusions after co-culture with 3T3-L1 cells. ***$p < 0.005$.

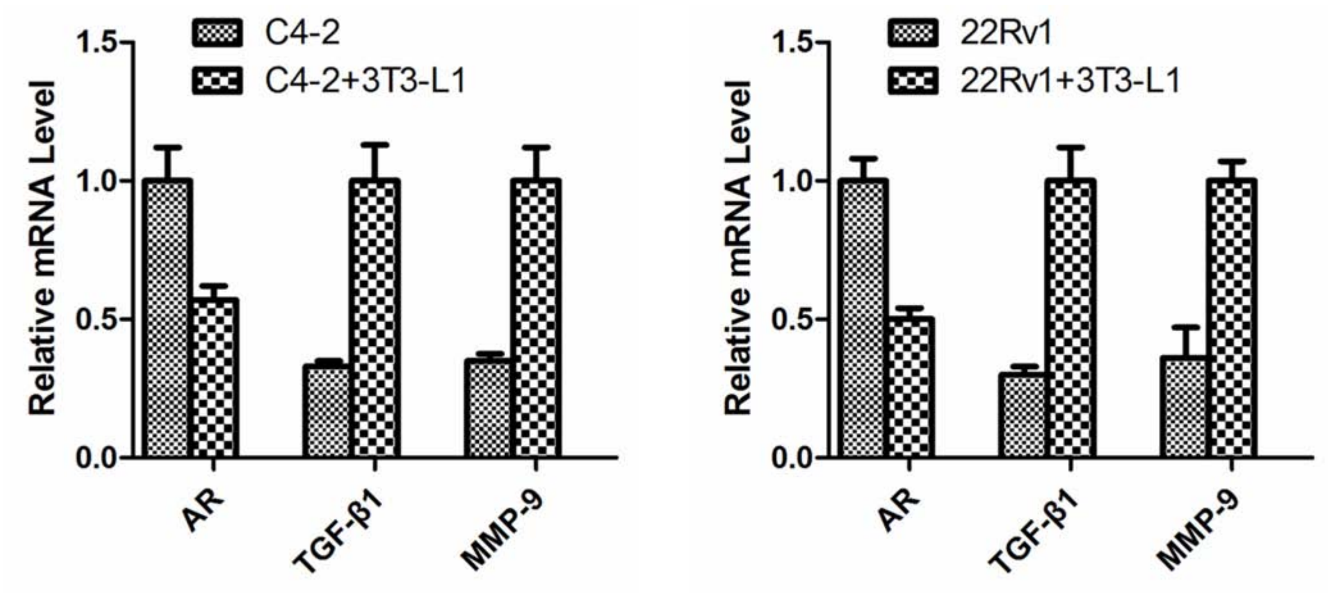

Supplementary Figure S4: mRNA level change of PCa cells after co-culture with mouse pre-adipocytes. mRNA level shows AR is down-regulated, TGF-β1 and MMP-9 are up-regulated after co-culture with mouse 3T3-L1 pre-adipocytes.

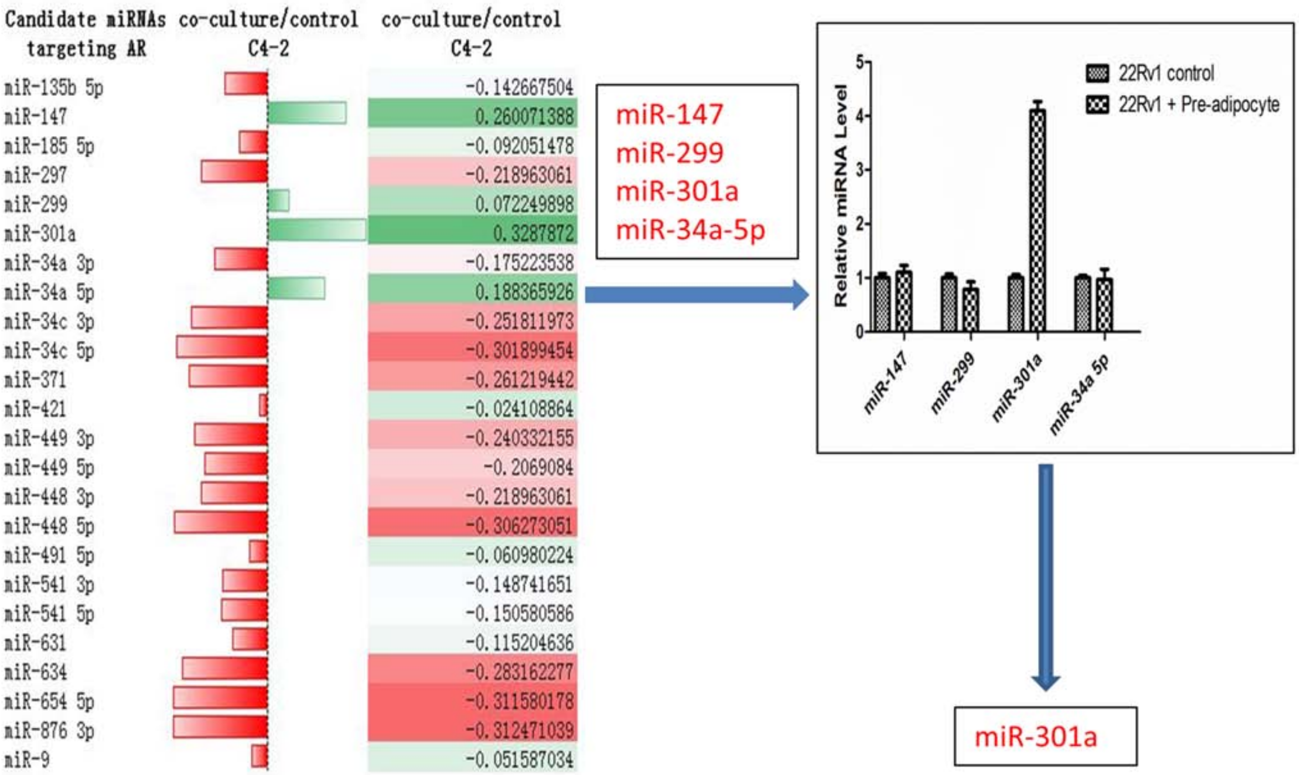

Supplementary Figure S5: miRNAs screen in PCa cells after co-culture with Pre-adipocytes. miRNAs that were predicted to target AR *via* website software were screened in C4-2 cells with and without co-culture with human pre-adipocytes, and the increased miRNAs were further confirmed in CWR22Rv1 (22Rv1) cells with and without co-culture with human pre-adipocytes.
